# High mobility, low access thwarts interventions among seasonal workers in the Greater Mekong Sub-region: lessons from the malaria containment project

**DOI:** 10.1186/s12936-016-1491-3

**Published:** 2016-08-26

**Authors:** Sara E. Canavati, Cesia E. Quintero, Harriet L. S. Lawford, Sovann Yok, Dysoley Lek, Jack S. Richards, Maxine Anne Whittaker

**Affiliations:** 1 Centre for Biomedical Research, Burnet Institute, Melbourne, Australia; 2 Department of Clinical Tropical Medicine, Faculty of Tropical Medicine, Mahidol University, 420/6 Ratchawithi Road, Ratchathewi, Bangkok, 10400 Thailand; 3 The National Centre For Parasitology, Entomology and Malaria Control, Ministry of Health, Corner Street 92, Trapaing Svay Village, Sankat Phnom Penh Thmey, Khan Sensok, Phnom Penh, Cambodia; 4 Provincial Health Department, Pailin City, Pailin Province Cambodia; 5 National Institute of Public Health, #2, St. 289, Toul Kork District, Phnom Penh, Cambodia; 6 Department of Medicine, University of Melbourne, Melbourne, Australia; 7 Department of Microbiology, Monash University, Melbourne, Australia; 8 College of Public Health, Medical and Veterinary Sciences, Division of Tropical Health and Medicine, James Cook University, Townsville, QLD 4811 Australia

**Keywords:** Community malaria worker, Artemisinin resistance, Cambodia, Malaria elimination, Health system strengthening, Mobile malaria worker, Sustainable development goals

## Abstract

**Background:**

During the process of malaria elimination in the Greater Mekong Sub-region, mobile and migrant populations (MMPs) have been identified as the most at-risk demographic. An important sub-group of MMPs are seasonal workers, and this paper presents an evaluation of the reach and effectiveness of interventions tailored towards this group and was carried out as part of the Containment Project from 2009–11.

**Methods:**

A mixed-methods study was conducted in Pailin Province in Western Cambodia. Three-hundred-and-four seasonal workers were surveyed using a structured questionnaire. Qualitative data were gathered through a total of eight focus group discussions and 14 in-depth interviews. Data triangulation of the qualitative and quantitative data was used during analysis.

**Results:**

High mobility and low access of the target population to the interventions, as well as lack of social and anthropological research that led to implementation oversights, resulted in under-exposure of seasonal workers to interventions. Consequently, their reach and impact were severely limited. Some services, particularly Mobile Malaria Workers, had the ability to significantly impact key factors, such as risky behaviours among those they did reach. Others, like Listening and Viewing Clubs and mass media campaigns, showed little impact.

**Conclusions:**

There is potential in two of the interventions assessed, but high mobility and inadequate exposure of seasonal workers to these interventions must be considered in the development and planning of future interventions to avoid investing in low-impact activities and ensure that all interventions perform according to their maximum potential. This will be critical in order for Cambodia to achieve its aim of malaria elimination. The lessons learned from this study can be extrapolated to other areas of health care in Cambodia and other countries in order to reduce the gap between healthcare provided to MMPs, especially seasonal workers, and to the general population.

## Background

### The vulnerability of mobile and migrant populations

In the final stages of elimination, the last foci of a disease is almost invariably found among vulnerable and marginalized demographic groups [1–4]. This is because these groups are systematically underserved, even by the world’s most well-resourced health systems. In order to “ensure the healthy lives and promote well-being for all at all ages” [5], as stated by Goal 3 of the Sustainable Development Goals, the unique needs of vulnerable and marginalized groups must be identified and confronted at the beginning of disease control and elimination efforts, and their broad range of health vulnerabilities must be addressed in a holistic and sustained manner.

During the process of malaria elimination in the Greater Mekong Sub-region, mobile and migrant populations (MMPs) have been identified as the most at-risk groups due to activities in which they engage, as well as the health system’s inability to reach them with routine surveillance and response strategies used for the general population [6]. The Cambodian Containment Surveys of 2009–10 found the prevalence of malaria among MMPs to be substantially higher than among the general population [7] and that MMPs were three times more likely to suffer clinical episodes of malaria [8]. This clearly indicates that despite Cambodia’s national goal of eliminating all forms of malaria by 2025, MMPs are much farther from this target than the general population. MMPs are regularly engaged in forestry and cross-border activities, and as a result often live and sleep in mosquito-ridden forests, placing them at substantially higher risk of infection [9]. There is also a lack of health services appropriate for their needs. A scarcity of tailored malaria education means that MMPs often do not know how to prevent or recognize malaria, or when and where to seek timely treatment [10]. Due to their high mobility, the existing health system is unable to provide adequate treatment and follow-up even to those who do seek help. The over-representation of malaria incidence among MMPs is merely symptomatic of their broader lack of access to public services and their increased vulnerability to many preventable health problems.

### Development of MMP profiles and intervention packages

A further barrier to addressing the needs of MMPs has been the fact that, although this is an extremely heterogeneous population, there are no clear definitions that will allow the identification of specific sub-group profiles with their particular risks and needs. In recognition of this gap, in 2012–2013 an MMP strategy that would enable malaria interventions to be tailored specifically to MMPs in Cambodia was undertaken by the National Malaria Control Programme and partners [11]. A standardized set of definitions appropriate to the Cambodian context was developed (Table 1), after which five different MMP profiles were identified based on a Population Movement Framework, MMP Activity Profiles, and a Malaria Risk Index, which is comprised of a vulnerability index, an exposure index and an access index [9]. Five strategic areas to be addressed were also identified: prevention, early diagnosis and treatment (EDAT), research, and surveillance, coordination and management [9]. Intervention packages that target each specific MMP profile while addressing each of these areas were then developed [9, 11]. An example of possible interventions per strategic area and MMP profile can be found in Table 2. It should be noted that a selection of these interventions were actually implemented in this study.

**Table 1:** Mobile migrant population (MMP) strategy definitions

Forest malaria	Malaria ecosystem and transmission is closely related to forested areas in Southeast Asia
Related activities of population	Population movement in relation to malaria, the main focus should be on the interaction/exposure of MMP with the forest
Local population	Permanent resident in the area for more than 1 year
Mobile population	Resident in the area for less than 6 months
Migrant population	Resident in the area for more than 6 months and less than 1 year
Visitors (from abroad to the country)	Person admitted for short stays for purposes of leisure, recreation, holidays; visits to friends or relatives; business or professional activities. Visitors include excursionists, tourists and business travellers. Tourists, visiting relatives who might spend one or two nights in or near the forest (e.g., family event, ecotourism)
Seasonal workers	Agricultural activities occurring during planting season (end of dry season) and harvesting season (end of rainy season), usually in foothills/plains/valleys. (e.g., farming/chamkar, rubber, cassava plantations)
Construction/mine workers	Activities related to infrastructure construction or mining in forested areas, usually in upland forest/hills/valleys. (e.g., dam or road construction, gold or gem mines)
Forest workers	Activities in heavily forested and remote areas in small mobile groups, usually in upland forest/hills. (e.g., forest products gathering, hunting, logging, fishing)
Security personnel	Activities related to patrolling in forested border areas

**Table 2:** Proposed interventions and delivery channels

Delivery mechanisms	Stage of journey	Strategic area	Categories of MMPs		
			Forest workers	Construction workers	Seasonal workers
			Retail sales (subsidized); voucher system	Lending scheme; retail sale; voucher system	Lending scheme; retail sale; voucher system
Behaviour change communication	Pre-departure en route upon arrival	Prevention	Mass media; taxi driver scheme	Mass media; IPC through MMWs; taxi driver scheme	Mass media; IPC through MMWs; taxi driver scheme
LLINs	Pre-departure en route upon arrival	Prevention	Forest package; taxi driver scheme	Forest package; taxi driver scheme; LLIN/ITN lending scheme	Forest package; taxi driver scheme; LLIN/ITN lending scheme
Diagnosis treatment	Upon arrival	EDAT	MMWs; stand-by treatment	MMWs; company health workers;	MMWs
MMP-surveillance	Upon arrival	Surveillance	Local authorities	Local authorities; company	Local authorities; plantation/farm owner/manager
MMP-malaria information systems	Upon arrival	Malaria surveillance	mHealth; private/public private mix	mHealth; private/PPM; MMWs	mHealth; private/PPM; MMWs

### Evaluation of interventions

Some of the interventions that were selected for the proposed intervention packages had already been implemented in Cambodia during the Containment Project [12], while others had not. This paper presents data from a mixed methods study that was undertaken in order to evaluate the effectiveness of prevention and EDAT interventions among seasonal workers in Pailin Province. MMPs who fit the ‘seasonal worker’ profile are a particularly prominent high-risk group in Pailin. Seasonal workers are engaged in agricultural activities during the planting and harvesting seasons, which take place at the end of the dry and rainy seasons, respectively. Usually they work in farms and plantations in foothills, plains and valleys; these include rice, corn and cassava farms, and rubber plantations (Table 2).

The interventions assessed in this study were designed to specifically target seasonal worker MMPs. Therefore, the study sites consisted exclusively of farms and plantations, where seasonal workers stay. This paper aims to examine the reach and effectiveness of these interventions. It is hoped that lessons learned from this study can help to improve these interventions, and allow the rest of the proposed interventions packages to be implemented more effectively among seasonal workers.

## Methods

### Interventions assessed

The following interventions were implemented in a Malaria Containment Project in Pailin Province, Western Cambodia in 2009–11: (1) a mass media education campaign broadcasted throughout areas of drug resistance was used to target MMPs. One film and several television and radio spots addressing malaria resistance, treatment and prevention were broadcast during what were believed to be peak viewing times [13, 14]. Listener and viewer clubs (LVCs) aimed to establish a collective ‘listening and/or viewing group’ that allowed participants to listen to or view programmes, and then actively engage in discussions. Mobile broadcasting units were set up in farms with no access to television or radio. Once a month, television and radio programmes were broadcast in the farm’s community centre, a space where workers often get together to socialize and drink after work. After the broadcast, the audience was engaged in a discussion and question and answer session [13, 14]; (3) an insecticide-treated net (ITN) lending scheme was implemented in farms where MMPs tended to work [15], as seasonal workers do not have access to the same free net-distribution scheme that villagers have. It empowered farm owners to lend ITNs to their employees and educate them on the benefits of using them; (4) taxi drivers in Palin Province were trained to provide malaria education and materials to MMPs and divert symptomatic passengers to a health facility upon obtaining their consent, due to the fact that 75 % of seasonal workers use taxis as their primary means of transportation [16]; and, (5) Mobile Malaria Workers (MMWs) were introduced in 2009 to provide health services to MMPs, as detailed elsewhere [17]. MMWs are a critical component of the national strategy to eliminate and contain drug-resistant malaria among MMPs in Cambodia. Among other things, they play a key role in providing EDAT to MMPs, including seasonal workers, who come to them seeking help for malaria symptoms. They also provide behaviour change communication to the MMPs and farm owners with whom they interact.

### Study team training and composition

Just prior to the fieldwork, a three-day workshop took place. Training was given on the purpose and exact procedures of the interviews and note taking, as well as on conducting interviews. In addition, a detailed guide with the standard operating procedures was provided to the field team. There were a total of three teams with two members per team. All tools were pre-tested in a community not already selected for the survey. A mixed-methods assessment of these interventions was conducted.

### Quantitative strand

#### Sampling

A cross-sectional study was carried out using a structured questionnaire. The target population of the study were seasonal workers at all MMW-assisted farms in Pailin Province. These farms had between 20 and 100 migrant workers in each farm. The MMW project did not target farms with less than 20 workers, so these farms could not be assessed in the study. At the time of the study, 28 MMW-assisted farms were actively operating in Pailin Province.

The required sample size was calculated using the main outcome indicator of the 2009 Containment Survey; a 60 % rate of long-lasting, insecticidal bed net (LLIN) use the night before the survey [8]. The sample size was, therefore, calculated to be 304 individuals based on an expected 60 % LLIN use, with a 5 % acceptable error, a type I error of 0.05 and a 10 % estimated non-response rate. The farm manager provided a list of names of seasonal workers in each farm, 80 % of whom were randomly selected to participate in the study; each farm was considered as one cluster; 304 individuals were selected with randomization at farm level. It included spouses in some cases, but no children were included.

#### Data analysis

Data were double-entered using an Epi Info^®^ database. Analysis was performed using Stata^®^ version 11 (StataCorp LP, College Station, TX, USA). Descriptive statistics, including basic frequencies and simple proportions, were calculated. The Mantel–Haenszel Chi square test or the Fisher’s exact test was used to calculate significance.

### Qualitative strand

#### Data collection and sampling

Qualitative data were gathered between December 2012 and January 2013 through eight focus group discussions (FGDs) (64 participants) and 14 in-depth interviews (IDIs) (14 participants) in two purposively selected farms. The data collection instruments were based on previously published methods [18] and on the Health Belief Model, and were further adapted throughout the study as necessary. Participants included MMWs, farm owners, seasonal workers, and taxi drivers. Purposive sampling was used to select participants to ensure gender distribution, a variety of ages, geographical provenances, intra-provincial travel capacities, and occupations.

#### FGDs

A total of eight FGDs were conducted among seasonal workers in MMW-assisted farms. Two farms in each health centre’s catchment area were purposively selected for geographical balance. As male and female workers have different risks and vulnerabilities, in each farm, two male and two female FGDs were conducted. Each FGD consisted of eight participants (N = 64).

#### IDIs

A total of 14 IDIs were conducted: three with farm owners, five with MMWs and six with taxi drivers. Farm owner and MMW IDIs took place at the same two farms as the FGDs. Taxi driver IDIs took place at the taxi stand in Pailin town.

#### Data analysis

FDGs and IDIs were recorded, fully transcribed, and translated into English. Data analysis consisted of examining, categorizing and tabulating or recombining the data. Thematic analysis around the key themes of the project was undertaken using Nvivo 9^®^ software. These themes were clustered to form overarching, larger themes. Triangulation and critical case analysis added rigour to the process.

### Ethical issues

Ethical clearance was obtained from the Cambodian National Ethics Committee for Health Research in August 2011 (130NECHR). Pailin local authorities, village chiefs, commune heads, and farm owners were informed of the purpose and expected duration of the study. Their approval and cooperation was sought in every aspect of data collection. For the quantitative strand, prior to the interview, the interviewer read carefully the consent form. This consent form contains information on the objectives of the survey, the risks, benefits and freedom of the participation, as well as information on confidentiality. Each survey participant provided informed written consent before participation. For the qualitative strand, the interviewers followed the Code of Ethics of the American Anthropological Association (AAA). As proposed by the AAA, all interviewees were informed before the start of the interview about the project’s goals, the topic and type of questions, the intended use of results for scientific publications, and their right to refuse the interview, interrupt the conversation at any time, and withdraw all given information during or after the interview. Anonymity was guaranteed and the confidentiality of interviewees assured by assigning a unique code number to each informant. Participants’ approval and cooperation was sought in every aspect of data collection.

## Results

### Demographic characteristics of seasonal worker study participants

All of the 304 seasonal workers who were approached for the study gave consent and participated in the survey. The average age of participants was 32 years, 55.6 % were males. The majority were farmers working in rice fields (48.7 %) and agricultural labourers working on other crops (49 %). Most had completed primary or secondary school or post-secondary education (57.9, 17.8 and 4.6 %, respectively), but 19.7 % had never attended school. The majority were married (66.1 %) (Table 3). The length of stay in farms was relatively short; for most seasonal workers it was less than a month (45.8 %), although others stayed for a longer period of around one or 2 months (39.4 %). More than half of the participants reported that it was their first time working in Pailin (Table 4).

**Table 3:** Sociodemographic characteristics of the seasonal workers

Sociodemographic characteristics	Total	
	N	%
Gender		
Male	169	55.6
Female	135	44.4
Age in years (mean, SD) [32, 10.6]		
Primary occupation		
Agricultural labourer	149	49.0
Seller	1	0.3
Fisherman	1	0.3
Forestry worker	2	0.7
Farmer	148	48.7
Housewife	1	0.3
Other	1	0.7
Highest level of education		
Never attended school	60	19.7
Completed primary (grade 6)	176	57.9
Completed secondary (grade 12)	54	17.8
More than secondary	14	4.6
Marital status		
Single, never married	89	29.3
Married/living with someone as married	201	66.1
Widowed	7	2.3
Divorced/separated	6	2.0
Married but not living together	1	0.3
Normally stay in the farm all year		
Yes	47	15.5
No	254	84.5
Accompanied by^a^		
Family	35	74.5
Friends	7	14.9
Alone	5	10.6

^a^Out of those who stay at the farm all year

**Table 4:** Frequency and duration of stay in Pailin farms

Seasonal worker characteristics	Total	
	N	%
First time working in Pailin		
Yes	166	54.6
No	138	45.4
Number of times worked in Pailin before (times)		
1–2	73	52.8
3–4	28	20.3
>5	37	22.3
Frequency of coming to work in Pailin		
Once a year	38	27.5
Twice a year	53	38.4
Three times a year	10	7.3
>3 times a year	14	10.1
I never leave	23	16.7
Length working in the farm (mean, SD) [18 weeks, 54] (weeks)		
<2	181	59.5
3–4	41	13.5
5–6	10	3.3
7–8	12	4.0
>8	60	19.7
Duration planned for this trip^a^ (months)		
<2	151	49.7
>2	152	50.3
Ways to find places of manual labour		
Friends	39	12.8
Farm owner	126	41.5
Used to come here before	23	7.5
Neighbour	109	35.9
Family member	73	24.0

^a^The 2-month cut-off point was defined as short-term migrants compared to those who stay for longer periods >2 months

### Evaluation of interventions

#### Education through mass media

Television was used more frequently than radio. Nevertheless, access to both radio and television was low. Even amongst participants who routinely consumed these media when not working at a farm, 54.7 % had not listened to the radio in the last month and 46.4 % had not watched television (Table 5). Only 22.1 % used radio and 25.4 % television on a daily basis while at the farm. This lack of access to media was reportedly due to a lack of time, reliable electricity, or access to a television set. Even though television was used more frequently than radio, more respondents had access to a radio set than to a television. Radio was a more common source of health messages despite being used less frequently. Children’s viewing habits were influential in determining the television programming of their parents. Respondents listened to the radio less in rainy (March/April) and harvest (July) seasons than at other times of the year. This observation was unexpected and requires further exploration in future studies. The study also found that the radio and television spots were not broadcast during this particular population’s peak viewing and listening times. National television channels were most often viewed between 17.00–18.00 and 21.00–22.00; key radio listening times were 06.00–0.7.00, 11.00–13.00 and 17.00–19.00 h.

**Table 5:** Media habits of seasonal workers in Pailin Province

Media habits	Overall	
	N	%
How often listen to the radio		
Once a week	12	6.6
2–4 times a week	26	14.4
5–6 times a week	4	2.2
Daily	40	22.1
Never	99	54.7
Last week, heard a message about malaria on the radio		
Yes	28	36.4
No	49	63.6
How often watch television		
Once a week	13	9.4
2–4 times a week	25	18.1
5–6 times a week	1	0.7
Daily	35	25.4
Never (during last month)	64	46.4
Last week, heard a message about malaria on television		
Yes	14	51.9
No	13	48.2

#### Listener and viewer clubs

Seasonal workers reported that the information presented through LVCs was interesting and easy to remember, as there were “many pictures”. Nevertheless, in general they were mostly ineffective at reaching seasonal workers; only 11.8 % of respondents had attended an LVC event. There was no significant increase in malaria knowledge among them. Seasonal workers had obtained the vast majority of their malaria-related knowledge from friends and family; official sources such as radio, television and MMWs were not predominant. Most of the malaria messaging reported by seasonal workers was inaccurate (Table 6).

**Table 6:** Communication and key messages reported by seasonal workers

Communication	Overall	
	N	%
Messages or information related to malaria prevention heard		
Malaria is caused by mosquito bites	258	84.9
Wear long sleeved clothes from dusk to dawn to prevent mosquito bites	121	39.8
Sleep under a mosquito net every night	175	57.6
Sleep under an insecticide-treated net	140	46.1
Buy the bundled net at the market and dip it with Super-Malatab	11	3.6
Super-Malatab is free, safe and easy to use	2	0.7
Malaria is dangerous	5	1.6
Malaria can kill	1	0.3
Messages related to malaria diagnosis and treatment heard		
Seek treatment for malaria from a MMW	202	66.5
Visit your MMW for free malaria diagnosis and treatment	39	12.8
Get a blood test before taking anti-malarial drugs	79	26.0
If you have fever, always seek a blood test for malaria at nearest health facility	59	14.4
Complete anti-malarial treatment	10	3.3
Do not buy cocktail; your malaria will not be cured	2	0.7
Messages related to using and caring for mosquito nets heard		
Sleeping under an ITN is especially important for pregnant women	2	0.7
Sleeping under an ITN is especially important for children under 5 years old	3	1.0
Carry and sleep under a mosquito net when travelling	92	30.3
Carry and sleep under a mosquito net when visiting the forest	28	9.2
The less you wash your treated net the longer it will retain its effectiveness	30	9.9
Repair any holes in the net	52	17.1
Do not use too much soap when washing your net	27	8.9
Dry mosquito net indoors	124	40.8
Prepare floor before tying	143	47.0
Source of messages		
MMW	68	22.4
Health facility staff	60	19.7
Private health provider/pharmacy	4	1.3
Teachers/religious leaders/monks	6	2.0
Family member/friend/neighbour	195	64.1
Television	81	26.6
Radio	88	29.0
Mobile video unit	5	1.6
Poster/leaflet/brochures/billboards	28	9.2
NGO staff	29	9.5
Have attended an event in the community with a screen/speaker on health messages		
Yes	36	11.8
No	268	88.2

#### Mobile Malaria Worker services

The majority of seasonal workers (71.1 %) did not know that an MMW was available for consultation in their region (Table 7), in large part because of the brevity of their stay there. As a result, they were the least common source of malaria education. MMWs were also the least common source for malaria education through non-media-related communication (15.6 %); the most common sources were family and friends (Table 8). In addition, MMWs were the least common source for seeking diagnosis when malaria infection was suspected (21.1 %) or obtaining drugs for malaria treatment (15.4 %). Seasonal workers instead sought care from other public health providers and private health providers (81.3 and 31.3 %, respectively) and obtained drugs from private health providers 46.2 % of the time (Table 9).

**Table 7:** Seasonal workers’ perceptions of Mobile Malaria Workers (MMWs)’ role

MMWs	Overall	
	N	%
Know the MMW		
Yes	88	29.0
No	216	71.1
MMW visited the migrants while in the farm^a^		
Yes	76	86.4
No	12	13.6
Received malaria education from MMW^a^		
Yes	81	92.0
No	7	8.0
Know that free malaria testing/treatment is available from MMWs		
Yes	84	95.4
No	4	4.5
Previously contacted MMW when having fever		
Yes	48	54.5
No	40	45.5
Ways migrants have contacted MMW		
Went to the MMW	59	67.1
MMW visited the migrant worker	16	18.2
Migrant worker never contacted MMW	13	14.8

^a^Out of those who have meet the MMW (n = 88)

**Table 8:** Interpersonal communication

Interpersonal communication	Overall	
	N	%
Ever discussed any topics concerning malaria		
Yes	218	71.7
No	86	28.3
Person with whom discussed malaria		
Family member	118	82.6
Villager	46	21.1
Friend	93	42.7
MMW	34	15.6
Topics discussed		
How to prevent malaria	163	74.8
How malaria is transmitted	102	46.8
How to treat malaria	53	24.3
Malaria drug resistance	4	1.8
Malaria testing	36	16.5
Where to seek advice	16	7.3
Who to seek advice from	4	1.8

**Table 9:** Options for seeking malaria treatment

Variable	Treatment sought at			
	MMW N (%)	Public health provider N (%)	Private health provider N (%)	Other N (%)
First action if a migrant thinks he/she has malaria	64 (21.1)	247 (81.3)	95 (31.3)	102 (33.6)^a^
Potential place visited for malaria test	64 (21.1)	263 (86.5)	89 (29.3)	–
Action taken if malaria test positive	54 (17.8)	271 (89.1)	113 (37.2)	14 (4.6)^b^
Action to be taken if febrile patient with negative malaria test	N/A	265 (87.17)	110 (36.18)	58 (19.1)^c^

Multiple answers by all interviewed migrants (n = 304) where possible

^a^Other includes take malaria test n = 86 (28.3 %); take drugs for malaria n = 16 (5.3 %)

^b^Other includes traditional medicine n = 4 (1.3 %); stay home n = 2 (0.7); self treatment n = 8 (2.6)

^c^Other includes stay home n = 7 (2.3); self-treat n = 43 (14.1); traditional medicine n = 8 (2.6)

A typical conversation during FGDs between seasonal workers and the moderator went as follows:

*M: Don’t you know that in the fields there are volunteers who help treat malaria?*

*P4: I don’t know because I’m just a new arrival.*

*M: How many days have you been here?*

*P4: 6* *days.*

*M: So, all of you, do you know that in the fields there are volunteers who help cure malaria fever?*

*P1: Don’t know.*

*P3: If we were living here for one or two years, surely we would know.*

*M: So, do you know, brother?*

*P6: No, I don’t.* […]

*M: So none of you here know that there are volunteers in the fields, do you?*

*P5: No!* (FGD 04 Seasonal workers)

The need for a more active mode of outreach to seasonal workers directly on the farms was a frequent subject of conversation among all participant categories. It was suggested that MMWs should post a sign or logo outside their home identifying themselves, so that seasonal workers and visitors know where to seek help. MMWs proposed going door-to-door to inform newcomers of their presence in the area.

Even when seasonal workers did come to MMWs for diagnosis, stock-outs often forced them to obtain anti-malarials in the private sector:

*…If the health centres did not have medicine, I wrote letters to malaria centre…so that patients could get medicine from the centre. If the centre had no medicine, I sent them to a private clinic.* (IDI 05, MMW, male).

MMWs reported that seasonal workers’ limited exposure to malaria education in their place of origin was a significant problem:

*The difficulty is that we cannot educate them as we educate villagers living with us. They don’t know much about methods of preventing malaria and about symptoms of malaria. Due to their lack of knowledge, migrants try to bear with the disease and think that it is a cold, and after a few days it becomes severe malaria.* (IDI 02, MMW, male).

MMWs also reported difficulties in providing quality care to seasonal workers, as the lack of access to transportation makes it difficult to transfer them to a health centre when they need specialized care, and their high mobility makes it nearly impossible to provide follow-up once treatment was started. Seasonal workers themselves agreed that their high mobility and lack of access was a significant barrier:

*There is no hospital around the farm. We have to go far away to Pailin city. Those who have worked here for a very long time know where to go for treatment because they have access to the village malaria worker, but for us, we don’t know where to go because we keep moving from one farm to another. Whenever we get sick, we go to state*-*run hospital in Pailin City or buy medicine at pharmacy.* (FGD 08, Seasonal workers).

Nevertheless, seasonal workers who received health services from MMWs were highly satisfied with the quality of the services provided in over 90 % of encounters (Table 10). MMWs were their most trusted source of malaria information, as they perceived them to be most knowledgeable. Close to no misconceptions on malaria knowledge were found among MMWs in qualitative interviews. Seasonal workers who had received health education from an MMW were 2.1 times more likely to report sleeping under an ITN the night before the survey (*P* = 0.002). Those who reported sleeping under an ITN the night before were 2.1 times more likely to discuss malaria-related topics with others (P = 0.006). Interpersonal communication (IPC) was the preferred method of malaria education (71.7 %) for seasonal workers.

**Table 10:** Reported satisfaction of services delivered by mobile malaria workers (MMWs)

Reported satisfaction of MMW services	Overall	
	N	%
MMW able to provide advice	82	93.2
Easy to get in touch with the MMW	82	93.2
Easy to communicate with the MMW	84	95.5
MMW understands malaria	79	90.1
MMW provides support (testing and treatment)	82	93.2
Satisfied by MMW services	84	95.5
Would refer an ill friend to MMW	84	95.5

MMWs themselves felt well accepted, and interviews showed that their services are appreciated:

*Volunteers don’t look down on us. When we go to see them, even though they are working, or stay far from us, they will come fast. There isn’t any problem.* (FGD 08, Seasonal workers).

It was the consensus among MMWs, farm owners and seasonal workers, that the work of MMWs is highly beneficial, and also provides a financial benefit:

*For me, I think it is very good that malaria volunteers come and teach about malaria prevention. As a farm owner, I can be aware of how I can help prevent my workers from being attacked by such a disease, and I can see many benefits. First, the workers working here are not sick, so they can speed up their work for me. Second, it is the workers’ benefit that they do not need to spend their money on medicines, and they can save some money to go back home. Next year they will work with me again since they can keep a good deal of money, won’t they?* (IDI 01, Farm owner).

*Migrant workers know that it is so beneficial when they come and get the medicine from me. If they are too ill to come, I can approach them. If they do not come to get the medicine from me, they have to spend up to 30,000 Riels (7.5* *US$) for the motor taxi.* (IDI 06, MMW, female).

#### LLIN/ITN lending scheme

The lending scheme had very high satisfaction rates. 98 % of seasonal workers who accessed the scheme were highly satisfied with the set-up; 97 % liked using an LLIN. 83 % had used the scheme in the past. Nevertheless, only 19.7 % of seasonal workers were found to have access to the LLIN lending scheme, as access very much dependent on the farm owners’ willingness to run the scheme. Some farm owners who did not participate in the lending scheme said they were too busy to take on this responsibility, or could not see what benefit it would bring them. Some declined to participate because “nets are meant to be free”. They also pointed to a lack of incentives that would compensate them for the additional effort invested in running the scheme.

Farm owners who implemented the scheme seemed to have an interest in protecting their labour force from malaria. When asked if they would consider buying nets for their workers if the lending scheme came to an end, a number of them said they would, as having healthier employees is more advantageous to them. Others said that they would be prepared to advance labourers’ wages to enable workers to buy nets from local markets at the beginning of their stay.

Seasonal workers currently enrolled in the scheme most often said that they had not brought a net with them because either their net was torn, they had been in a great hurry when migrating, or they did not have the money to buy one. Only 50 % of seasonal workers currently enrolled in the scheme were willing to pay out of their own pockets for a net if the scheme came to an end.

#### Taxi driver scheme

Taxi drivers indicated that the satisfaction they gained from playing a leadership role and protecting others from malaria infection were their main motivators for volunteering in the scheme. They said that they referred clients who were ill with fever to a hospital or MMW, and transported them there whenever possible. Although the majority of taxi drivers displayed competent malaria knowledge, particularly with regard to recognizing symptoms and accessing malaria services, many had crucial knowledge gaps regarding matters such as the role of mosquitoes in the spread of malaria and effective use of bed nets. Many drivers themselves did not use ITNs, instead opting for untreated nets.

## Discussion

This is the first published study assessing the effectiveness of malaria interventions tailored specifically to seasonal workers in Pailin Province, the global epicentre of multidrug-resistant malaria. The study found that that even though these interventions were specifically designed for seasonal workers, they largely failed to have the expected impact because they were not accessible to their target population, or because seasonal workers were too mobile and did not remain in the area long enough to be impacted by continual exposure to the interventions. MMW services and the LLIN lending scheme were the most successful interventions for seasonal workers in Cambodia but overall their impact was limited due to low access. Similar issues have found to affect other health services being provided to MMPs [19, 20].

### Mass media and LVCs

Behaviour change communication strategies have been highly successful in Cambodia among the general population [21], and it is natural to seek to expand those messages using mass media strategies. Nevertheless, major constraints to effective use of mass media in this study included limited access to radio and television sets, and the discrepancy between peak viewing times of seasonal workers and the times during which radio and television malaria spots were broadcast. LVCs were also largely unsuccessful. This is likely because seasonal workers proved to be too highly mobile to be reached effectively and consistently by them; the average seasonal worker spent less than 1 month at the farm, and LVCs were held only once a month. It was also noted that workers who intended to consume alcohol and unwind often frequented the meeting places where LVCs were held, therefore making it a less effective environment for educational activities.

### MMW services

Seasonal workers unanimously rated the quality of services provided by MMWs as exceptionally high on all counts and MMWs could be extremely effective in combating malaria if they were able to have face-to-face contact with seasonal workers. MMWs provided health messages through IPC, the preferred method of education for seasonal workers. When they did so, they became the most trusted source of information and were reportedly successful in modifying risky behaviours and disseminating malaria education. A recent study has documented that IPC is the most successful form of behaviour change communication in Western Cambodia [21]. MMWs in this study had very few malaria-related misconceptions, which was not the case for the main village malaria workforce in a recent assessment [17]. They were therefore an accurate source of IPC that, with sufficient exposure, could successfully counter the misconceptions that this study found were disseminated through the most common source of malaria education: friends and family. MMWs were also well received by both farm owners and workers, even viewed as an economic advantage to both. It is clear that they were effective, reliable and ideally suited to providing both prevention and EDAT to seasonal workers. It is therefore highly disappointing that MMWs were the least-accessed source of both education and malaria services due to a lack of awareness and access. The MMW programme has enormous potential that can be leveraged by pro-actively reaching out to seasonal workers immediately after their arrival. MMWs should employ marketing techniques to make their presence known in the area and should encourage community participation in prevention practices, as seen in other settings [22–26]. Lessons learned from village malaria workers and community health workers to improve motivation, satisfaction, barriers to follow-up, scale-up, expanding activities, and improving the effectiveness of interventions can be applied to MMWs [17, 27–35]. Of concern was the fact that a large proportion of seasonal workers sought treatment in the private sector, despite evidence that the private sector often treats malaria with counterfeit or sub-standard drugs that are a major driver of evolving multidrug-resistance in the area [36]. It has also been documented that stock-outs, as reported by MMWs in this study, are a major driver of private sector health-seeking among MMPs [17]. It is critical to resolve supply chain inefficiencies.

### LLIN/ITN lending scheme

The ITN lending scheme demonstrated a significant level of success in addressing the lack of availability of ITNs among seasonal workers, and enjoyed a 98 % satisfaction rate. Nevertheless, with only 20 % of seasonal workers accessing it, it is clear that the scheme would need to be scaled-up significantly in order for it to be effective. Educating farm owners on the financial benefits of a healthy workforce, and providing incentives for launching the scheme should persuade more farm owners to implement it. However, it is essential that a plan for sustaining the scheme be developed prior to scale-up, as another study found that between 2011 and 2013, 16 % of loaned ITNs were broken, 24 % were lost and approximately 40 % were not returned [15]. In an unpublished respondent-driven sampling study conducted among migrant workers in Western Cambodia while this lending scheme was ongoing, a much higher proportion of migrant workers slept under a bed net in Pailin than in a neighbouring province, and the number of migrant workers that slept under a borrowed net was double that of the other province [37]. This suggests that the lending scheme made a noticeable positive impact.

### Taxi driver scheme

The taxi driver scheme showed promising results due to high motivation from the drivers, and the added convenience of being able to transport patients to a health facility immediately. The inadequate knowledge of malaria-related subjects for some taxi drivers however, was concerning and presents many opportunities for improvement. A reminder sheet with basic facts and details could serve to support the delivery and consistency of key messages. The scheme could also be expanded to buses in order to target seasonal workers using cheaper forms of transportation, as they are likely to be even more economically disadvantaged [38]. Bus and taxi drivers could also be provided with ITNs to sell for a very small profit.

### Study limitations

Potential limitations of the study relate to the self-reporting nature of the data and the lack of a comparison to actual observed behaviours. It is also important to note that some of the responses given by seasonal workers to MMWs may have been biased by the desire to provide socially acceptable answers (i.e., seasonal workers who had been in contact with an MMW were 2.1 times more likely to report sleeping under a bed net; this suggests that they had received malaria messages from the MMW that either had increased their knowledge and therefore alerted them to the social desirability of this answer, or successfully modified their behaviour. It is difficult to know the proportions of each). A further possible limitation relates to the high-mobility of the seasonal workers and the likelihood that this limited the duration of interventions and therefore their potential impact. This however, is the nature of seasonal workers lives and reflects the difficulty in implementing long-term interventions for this group.

### The need for future social and anthropological research

In future, rigorous social and anthropological research should be undertaken when planning new interventions [39]. In doing so, it is crucial to remember that MMPs are a highly heterogeneous demographic, and what applies to one group may not apply to another [9, 40]. The use of social and anthropological research to more clearly define MMP profiles can then be used to tailor interventions more effectively (Table 11). For example, MMPs’ frequent travels between endemic and non-endemic areas are a major factor in their vulnerability to malaria, and anthropological studies have helped to tailor specific interventions to different MMP sub-groups during the various stages of migration (Table 2) [9, 41, 42]. Similarly, in this study, MMWs reported that seasonal workers from non-endemic areas had increased chances of infection by failing to protect themselves because of a lack of being exposed to basic malaria education, an issue that could be addressed by focused pre-departure interventions.

**Table 11:** Summary of mobile migrant population profiles (Adapted from Guyant et al. [9])

	Variables	Forest workers	Construction workers	Security personnel	Seasonal workers	Visitors
Profile		FW	CW	SP	SW	T
	Main activities	Hunting, fishing, logging, non-timber forest products	Dam or road construction, mining	Patrolling	Farming, plantation, *chamkar*	
	Population type	Local, Mobile, Migrant	Mobile, Migrant	Mobile, Migrant	Local, Mobile, Migrant	Mobile
Forest/malaria exposure	Location from forest	In forest	In forest/forest fringe	In forest	Forest fringe	Forest fringe
	Duration of stay in forest	1–4 weeks	1–6 months	Weeks to months?	1–4 weeks	1 week
	Forest exposure	High	Medium to high	High	Low to medium	Low
	Housing type	Tents, none	Huts, barracks, tents	Huts, barracks, tents	Tents, huts	Wooden or concrete house
Working conditions/access/outreach	Work area	Forest, hills	Forest, hills	Border forest	Foot hills, plains, valleys	
	Work location	Mobile	Fixed	Semi-mobile	Fixed	
	Link/affiliation	None or village for local population	Company	Government	Farm owner/company	None
	Main point of contact	None or village for local population	Company	Military base	Farm owner/company	Guest houses/hotels

## Conclusions

The study found that there was real potential in two out of five of the interventions assessed, although longer term sustainability was not evaluated. Nevertheless, low access and high mobility of seasonal workers affected the degree of exposure they had to the interventions, and therefore the degree to which they could be impacted by them. These two factors must be accounted for in the development and planning of future interventions to avoid investing in low-impact activities and ensure that all interventions perform according to their maximum potential. The lack of access is a barrier that must be overcome if malaria elimination is to be achieved. Lessons learned from this study can be extrapolated to other areas of health care, with the hope that they can reduce the gap between health care provided to MMPs and to the general population.

## Abbreviations

EDAT: early diagnosis and treatment
FGD: focus group discussion
IDI: in-depth interview
IPC: interpersonal communication
ITN: insecticide-treated net
LLIN: long-lasting, insecticidal net
LVCs: listener and viewer clubs
MMWs: mobile malaria workers
MMPs: mobile and migrant populations
